# Intrinsic N‑Terminal Reactivity and Improved Analysis of DSSO-Carbamate and Carbamate-Based Cross-Linkers

**DOI:** 10.1021/acs.analchem.5c06834

**Published:** 2026-03-11

**Authors:** Alessio Di Ianni, Thomas Fabian Leischner, Bogdan-Razvan Brutiu, Iakovos Saridakis, Andrea Di Ianni, Hendrik Krolle, Christian H. Ihling, Saad Shaaban, Nuno Maulide, Andrea Sinz, Claudio Iacobucci

**Affiliations:** † Center for Structural Mass Spectrometry, 9176 Martin Luther University Halle-Wittenberg, Kurt-Mothes-Str. 3, Halle/Saale D-06120, Germany; ‡ Department of Pharmaceutical Chemistry & Bioanalytics, Institute of Pharmacy, 9176 Martin Luther University Halle-Wittenberg, Kurt-Mothes-Str. 3, Halle/Saale D-06120, Germany; § Human Technopole, V.le Rita Levi Montalcini 1, Milan 20157, Italy; ∥ Institute of Organic Chemistry, 27258 University of Vienna, Wien 1090, Austria; ⊥ Molecular Biotechnology Center, Department of Molecular Biotechnology and Health Sciences, University of Turin, Turin 10126, Italy; # NBE-DMPK Innovative BioAnalytics, Merck Serono RBM S.p.A., an Affiliate of Merck KGaA, Darmstadt, Germany, Via Ribes 1, Colleretto Giacosa, Turin (TO) 10010, Italy; ∇ MS Vision, Televisieweg 40, Almere 1322 AW, The Netherlands; ○ Division of Bioanalytical Chemistry, Vrije Universiteit Amsterdam, De Boelelaan 1105, Amsterdam 1081 HV, The Netherlands; ◆ Centre for Analytical Sciences Amsterdam, Amsterdam 1098 XH, The Netherlands; ¶ Department of Physical and Chemical Sciences, 9303 University of L’Aquila, Via Vetoio, L’Aquila 67100, Italy

## Abstract

Cross-linking mass spectrometry (XL-MS) has emerged as a powerful approach for probing protein structure and conformational dynamics. Conventional cross-linkers typically contain two *N*-hydroxysuccinimide (NHS) ester groups that primarily target lysine residues. Here, we report the optimization of the in-solution reactivity of disuccinimidyl sulfoxide carbamate (DSSO-carbamate), an analogue of DSSO in which the two NHS ester groups are replaced by NHS carbamates. The enhanced stability of the carbamate functionality reduces the degradation of DSSO through retro-ene sulfoxide elimination under standard XL-MS buffer conditions, thereby improving cross-linking efficiency. We further characterized the gas-phase dissociation behavior of DSSO-carbamate and optimized the collision energy (CE) parameters for automated data analysis with XL-MS search engines. Mapping of cross-linking sites for bovine serum albumin revealed an unexpectedly high frequency of cross-links involving the protein N-terminus, suggesting increased N-terminal reactivity of NHS carbamates relative to NHS esters. This hypothesis was corroborated by comparative cross-linking of nonacetylated and N-terminally acetylated α-synuclein using DSSO-carbamate and the NHS ester-based disuccinimidyl dibutyric urea (DSBU). We observed the same reactivity trend for the NHS carbamate-based cross-linker NNP9. Proteome-wide XL-MS analysis confirmed a higher propensity of NHS carbamate-based reagents to form cross-links with protein N-termini compared to NHS ester-based cross-linkers. Together, these results show that NHS carbamate-based reagents provide complementary XL-MS restraints to NHS ester-based cross-linkers and are particularly useful for investigating systems where N-terminal interactions are functionally relevant. We anticipate that this unique N-terminal selectivity of NHS carbamates will find broader applications in bioconjugation and chemical proteomics.

## Graphical abstract

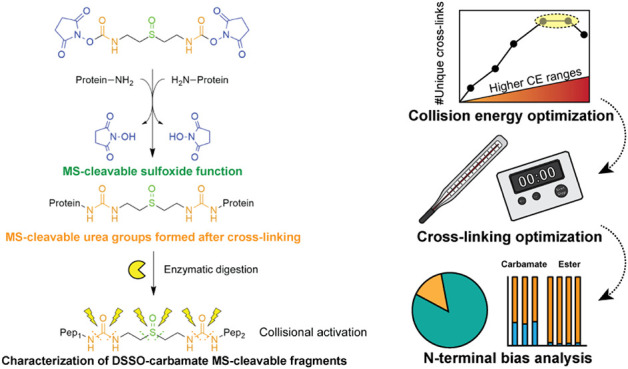

## Introduction

Over the past decade, numerous cross-linking reagents have been developed by different research groups.^1–12^ Initially, homobifunctional, noncleavable, and amine-reactive *N*-hydroxysuccinimide (NHS) esters, such as disuccinimidyl suberate (DSS) or its water-soluble sulfonic acid analogue (BS3),^13,14^ were most commonly used. These reagents predominantly react with lysine residues and, to a lesser extent, with protein N-termini and hydroxyl-containing amino acids. Later, novel chemistries were developed to target other amino acids in either a specific or unspecific fashion. Specific warheads include haloacetamide or maleimide moieties for cysteines,^9,15^ carbodiimides or triazines for amine–carboxyl coupling,^16,17^ and hydrazides for carboxylic acids.^16,18^ Unspecific XL-MS reactive groups generally rely on photocross-linking principles; examples include phenyl azides, benzophenones, and alkyl diazirines.^19–21^ Modern MS-cleavable cross-linkers based on NHS ester chemistry are also available. Early examples include the protein interaction reporter (PIR),^1^ disuccinimidyl dibutyric urea (DSBU),^2^ and disuccinimidyl sulfoxide (DSSO),^3^ which became commercially available in the past decade. MS-cleavable cross-linkers dissociate in the gas phase, revealing the masses of the two individual cross-linked peptides and thus reducing the otherwise quadratic data search space to a linear problem.^22^ This feature greatly accelerates XL-MS data analysis, making such reagents ideal for proteome-wide applications. PIR, DSBU, and DSSO represent the progenitors of the Rink-, urea-, and sulfoxide-based classes of MS-cleavable reagents, which have been further refined over the years. Nowadays, XL–MS has expanded well beyond purified proteins and reconstituted assemblies, enabling the interrogation of highly complex biological systems such as cell lysates and even intact cells.^12,23,24^ In this context, proteome-wide XL–MS can provide valuable structural insights into protein–protein interactions and the architecture of protein complexes directly in their native environment, becoming increasingly adopted as a powerful tool in structural biology. As cross-linking efficiency decreases substantially in highly complex systems,^25^ cross-link enrichment becomes essential to increase the depth of XL–MS analysis. Chromatographic fractionation approaches, such as size exclusion chromatography (SEC) or strong cation exchange (SCX), either alone or combined with affinity-based purification,^26,27^ represent robust strategies to reduce sample complexity and improve cross-link identification. For instance, cross-links can be enriched via an installed biotin handle (e.g., the Leiker reagent^28^) or by click-chemistry workflows, as exemplified by azide/alkyne-functionalized disuccinimidyl bissulfoxide (DSBSO) cross-linkers.^29^ Additional enrichment strategies include IMAC-based isolation of phosphonic acid–tagged cross-links (e.g., PhoX and tBuPhoX^23,30^ ) and, more recently, antibody-based enrichment enabled by tandem mass tagging (TMT), such as the 2,6-dimethylpiperidine disuccinimidyl tridecanoate (DPST) cross-linker.^31^ Notably, most enrichment-enabled reagents reported to date are homobifunctional and rely on NHS ester chemistry. Therefore, NHS ester-based cross-linkers remain the most widely used reagents in XL-MS studies. The half-life of NHS esters in aqueous solutions ranges from minutes to hours due to their high chemical reactivity. On one hand, this ensures efficient protein cross-linking; on the other, a large proportion of the cross-linker reacts with water, forming mono-links and dihydrolyzed inactive products. As an alternative, NHS carbamates represent a promising solution. In 2015, Nury and coworkers introduced a trifunctional cross-linker, NNP9, featuring two reactive carbamate moieties.^32^ Carbamates are less prone to hydrolysis than esters and generally less reactive.^33,34^ This reduced reactivity results from resonance stabilization of the carbonyl carbon by the carbamic nitrogen atom, which lowers its electrophilicity. Increased delocalization of π-electrons also makes carbamates more rigid than the corresponding esters. Despite their lower reactivity, it has been proposed that the slower hydrolysis rate is advantageous for increasing the yield of intra- and intermolecular protein cross-links. Faustino and coworkers later introduced NHS carbamate functionalities into the DizSEC and DizSPC cross-linkers.^35^ Incorporation of carbamate groups can also stabilize labile cross-linker backbones that are otherwise prone to decomposition. For example, Saridakis and coworkers incorporated two carbamate groups into the DSSO backbone, which resulted in higher stability in anhydrous dimethyl sulfoxide (DMSO) compared to NHS esters, thereby reducing DSSO decomposition via retro-ene sulfoxide elimination.^36^ Moreover, the lower electrophilicity of the carbamate reagent improved the recovery of mono-linked species, which can provide additional structural information.

In this work, we studied in detail the recently introduced DSSO-carbamate cross-linker.^36^ We optimized its in-solution reaction conditions using bovine serum albumin (BSA) as a model protein and characterized its peculiar gas-phase dissociation behavior under different collision energy regimes to improve automated cross-link analysis. Interestingly, a remarkable fraction of BSA cross-links involved the protein N-terminus, suggesting a higher propensity of the carbamate moiety to target protein N-termini, in contrast to NHS esters. To confirm this finding, we compared DSSO-carbamate with the NHS ester-based urea cross-linker DSBU using both nonacetylated and N-terminally acetylated α-synuclein. For the acetylated form, DSSO-carbamate cross-linking was largely suppressed, whereas DSBU reactivity was only mildly affected. Finally, we investigated whether the observed N-terminal selectivity of NHS carbamates was specific to DSSO-carbamate or represented a general feature of carbamate-based cross-linkers. To this end, we employed NNP9 to cross-link an antibody–antigen complex in which N-terminal binding of the antibody’s light chain plays a key role. Also in this case, the NHS carbamate-based NNP9 reagent preferentially cross-linked the protein N-terminus. Ultimately, we also recapitulated the complementarity of NHS carbamate-based cross-linkers in proteome-wide XL-MS. Altogether, our data reveal that NHS carbamates display a distinct selectivity toward protein N-termini. This finding is relevant for protein cross-linking, as NHS carbamate-based cross-linkers offer complementary structural restraints to classical NHS ester reagents. Moreover, this N-terminal selectivity may have broader implications for protein functionalization, including bioconjugation, chemical proteomics, and covalent drug design.

## Experimental Section

### Materials

All chemicals used in this study were obtained from Merck. Trypsin (porcine, sequencing grade) was obtained from Promega. LC-MS grade Nano-HPLC solvents were acquired from HiPerSolv CHROMANORM, VWR. Bovine serum albumin (BSA) was obtained from Sigma. Disuccinimidyl dibutyric urea (DSBU) cross-linker was purchased from CF Plus Chemicals, NNP9 cross-linker was acquired from Glixxlabs. The α-syn gene was ligated into a pET21a­(+) vector using the XhoI and NdeI restriction sites. The pTSAra-NatB plasmid was kindly provided by Tim Bartels (Harvard Medical School) for coexpression. Human recombinant TNFα (10602-HNAE) was purchased from Sino Biological, full-length Infliximab biosimilar (SIM0006) was acquired from BioXcell.

### Methods

#### Synthesis of DSSO-Carbamate

DSSO-carbamate was synthesized according to a published protocol.^36^

#### Cross-Linking Experiments

DSSO-carbamate *reactivity tests on bovine serum albumin.*

Bovine serum albumin (BSA) was resuspended in 50 mM HEPES buffer, pH 7.4 at a concentration of 10 μM (0.66 μg/μL). DSSO-carbamate was freshly dissolved in neat dimethylsulfoxide (DMSO) and added to the protein solution to a final concentration of 1 mM. To optimize cross-linking yield, two experimental variables were changed (4 different cross-linking reactions):

- Reaction time: 1 h, 2 h
- Temperature: room temperature and 37 °C

All reactions were quenched using ammonium bicarbonate (ABC) at a final 50 mM concentration for 10 min.

##### α-syn and N-Terminally Acetylated α-syn Cross-Linking

α-syn and N-terminally acetylated α-syn (NAc_α-syn) were expressed and purified according to a published protocol.^37^ α-syn and NAc_α-syn were cross-linked at 50 μM in phosphate buffer, pH 7.4 using DSSO-carbamate or DSBU at 2-fold or 10-fold molar cross-linker excess for 1 h at room temperature. Cross-linkers were freshly dissolved in neat DMSO prior to addition to the protein solution. Reactions were quenched using ABC at a final 50 mM concentration for 10 min.

##### Infliximab-TNFα NNP9 Cross-Linking

Human recombinant TNFα (20 μM) and full-length Infliximab biosimilar (10 μM) were incubated at room temperature for 60 min prior to triggering the cross-linking reactions. Phosphate-Buffered Saline (PBS, pH 7.4) was used as reaction buffer for NNP9. NNP9 cross-linker was added to the protein mixture to a final concentration of 0.5 mM and 1 mM (respectively 50- and 100-fold excess over the antibody concentration). The cross-linker was dissolved in neat DMSO at a concentration of 25 mM immediately before adding it to the protein solution. The solution was incubated at room temperature for 60 min and quenched with ABC (40 mM final concentration) for 15 min.

For all XL-MS experiments, the final DMSO concentration was 2% v/v to not perturb protein structure.

#### Sample Processing and Digestion

##### In-Solution Digestion of Cross-Linked BSA Samples

Cross-linked BSA samples were denatured using urea (6 M final concentration), reduced with 45 mM dithiothreitol (DTT), and alkylated with 100 mM iodoacetamide (IAA). Samples were then diluted to 1 M urea before adding trypsin (20:1 protein to enzyme ratio) for a total digestion time of 16 h. The proteolysis was quenched with trifluoroacetic acid (TFA) by lowering the pH to ∼2.

##### Sodium Dodecyl Sulfate Polyacrylamide Gel Electrophoresis of NNP9 Cross-Linked Samples

Samples were resolved using sodium dodecyl sulfate polyacrylamide gel electrophoresis (SDS-PAGE). In detail, samples were loaded on 10% precasted polyacrylamide mini gels (NuPAGE 10%, Bis–Tris, 1.0–1.5 mm, Invitrogen, Thermo Fisher Scientific). Cross-linked samples were denatured using Pierce Sample Loading Buffer (1×) (Invitrogen, Thermo Fisher Scientific) in the presence of lithium dodecyl sulfate (LDS). Samples were heated up on a preheated thermomixer at 70 °C/1100 RPM for 10 min before gel loading. 2-(*N*-Morpholino)­ethanesulfonic acid (MES) SDS Running Buffer (Invitrogen, Thermo Fisher Scientific) was used at a final 1× concentration as running buffer. The electrophoretic run was performed at a constant voltage of 200 V for 30 min. A ready-to-use Coomassie G-250 stain (SimplyBlue SafeStain, Invitrogen, Thermo Fisher Scientific) was used for 2 h at 37 °C on a shaker for visualizing protein bands on the polyacrylamide gel.

##### Enzymatic In-Gel Digestion of NNP9 Cross-Linked Samples

Gel bands corresponding to antibody–antigen complexes were excised from the gel and introduced in Protein LoBind Eppendorf tubes. Bands were dried in acetonitrile, reduced with 10 mM DTT in 50 mM ABC for 30 min at 60 °C. Tubes were cooled down to room temperature before the alkylation step using 55 mM IAA solution in 50 mM ABC for 20 min in the dark at room temperature. Trypsin digestion (protein to enzyme ratio 20:1) using freshly prepared trypsin was performed at 37 °C overnight, followed by chymotrypsin digestion for 4 h at 37 °C. Peptides were recovered using an extraction buffer (5% v/v TFA/acetonitrile 1:2). The obtained peptide mixture was evaporated in a Genevac evaporator, and the final dried samples were resuspended in an aqueous solution containing 2% v/v acetonitrile in 0.1% v/v TFA and subjected to liquid chromatography tandem mass spectrometry (LC-MS/MS) analysis.

#### Mass Spectrometry

##### Nano-HPLC/Nano-ESI-MS/MS Analysis of DSSO-Carbamate Cross-Linked Samples

DSSO-carbamate cross-linked BSA peptides were analyzed by LC-MS/MS on an UltiMate 3000 RSLC nano-HPLC system (Thermo Fisher Scientific) coupled to an Orbitrap Fusion tribrid mass spectrometer equipped with an Easy-Spray ion source (Thermo Fisher Scientific). Peptides were trapped on a C18 precolumn (Acclaim PepMap 100, 300 μm × 5 mm, 5 μm, 100 Å, Thermo Fisher Scientific) and separated on a Easy-Spray C18, 50 cm column (Thermo Fisher Scientific). After trapping, peptides were eluted by a concave 90 min water-acetonitrile gradient from 3 to 30% B (solvent B: acetonitrile, 0.1% formic acid) at a flow rate of 300 nL/min. Data were acquired in data-dependent MS/MS mode using several stepped higher energy collision induced dissociation (stepped-HCD) settings. In detail, collision energy screening was carried out using 18–21–24, 21–24–27, 24–27–30, 27–30–33 NCE%. High-resolution full scans (*m*/*z* 300–1700, *R* = 120,000 at *m*/*z* 200) were followed by high-resolution product ion scans (*R* = 15,000) for 5 s, starting with the most intense signal in the full scan mass spectrum. Precursor ions with charge states >2+ and <8+ were selected for fragmentation; the isolation window was set to 2 Th. Dynamic exclusion of 60 s was enabled to allow the detection of less abundant ions.

##### Nano-HPLC/Nano-ESI-MS/MS Analysis of NNP9 Cross-Linked Samples

NNP9 cross-linked samples were analyzed by LC-MS/MS on an UltiMate 3000 RSLC nano-HPLC system (Thermo Fisher Scientific) coupled to a Q-Exactive Plus Orbitrap mass spectrometer (Thermo Fisher Scientific) equipped with an Easy-Spray ion source (Thermo Fisher Scientific). Peptides were trapped on a C18 precolumn (Acclaim PepMap 100, 300 μm × 5 mm, 5 μm, 100 Å, Thermo Fisher Scientific) and separated using a C18 reversed phase column set at 35 °C (75 μm i.d. × 150 mm, Pep Map RSLC C18, 2 μm, 100 Å, Thermo Fisher Scientific). After trapping, peptides were eluted at a flow rate of 400 nL/min using a linear 96 min gradient of 4–95% B, followed by a 10 min column wash at 95%B, and re-equilibration for 24 min (solvent A: 0.1% formic acid in water; solvent B: 0.1% formic acid in acetonitrile). Cross-linked peptides were analyzed by LC-MS/MS; in detail, high-resolution full scans (*m*/*z* 200–1500, *R* = 70,000 at *m*/*z* 200) were recorded in the orbitrap mass analyzer. Data-dependent high-resolution product ion scans of the 10 most abundant ions were recorded in the orbitrap (*R* = 35,000 at *m*/*z* 200), starting with the most intense signal in the full scan mass spectrum. Normalized stepped higher-energy collision induced dissociation (HCD) of 26, 30, 34 V was applied. Precursor ions with charge states >2+ and < 8+ were selected for fragmentation; the isolation window was set to 2 Th. Dynamic exclusion of 60 s was enabled to allow detection of less abundant ions.

##### Native Mass Spectrometry of Cross-Linked α-syn and NAc_α-syn

Samples were analyzed via native mass spectrometry (MS) with a high-mass Q-TOF II mass spectrometer (Micromass/MS Vision). Protein solutions were buffer-exchanged 8 times in ammonium acetate (500 mM, pH 6.8) using centrifugal filtration units with 3 kDa molecular weight cutoff (MWCO). Sample volumes used for all the native MS experiments were 5 μL. Samples were ionized by nano-ESI using in-house, gold-coated borosilicate glass capillaries. Capillary voltage was set to 1.2–1.4 kV and the cone voltage to 90–120 V. The pressure in the source area of the MS (p1) was set to 10 mbar and the pressure in the collision cell (p3) was set to 1 × 10^–2^ mbar. Cesium iodide (CsI) measurements were carried out for the calibration of the mass spectra.

#### Data Analysis

Identification of cross-links was performed with MeroX software.^38,39^ MeroX settings for all the different samples are reported in the Supporting Information file.

## Results and Discussion

### Fragmentation Behavior and Collision Energy Optimization of DSSO-Carbamate Cross-Links

DSSO-carbamate represents an addition to the arsenal of MS-cleavable cross-linkers developed thus far. Compared to DSSO, DSSO-carbamate exhibits slightly higher polarity due to the presence of two additional nitrogen atoms (Figure S1). Despite this modification, its topological polar surface area (tPSA) remains comparable to that of its membrane-permeable predecessor. Like DSSO, DSSO-carbamate features a central sulfoxide moiety that generates characteristic fragment ions for each cross-linked peptide upon higher-energy collisional dissociation (HCD) (Figure 1).

**Figure 1:**
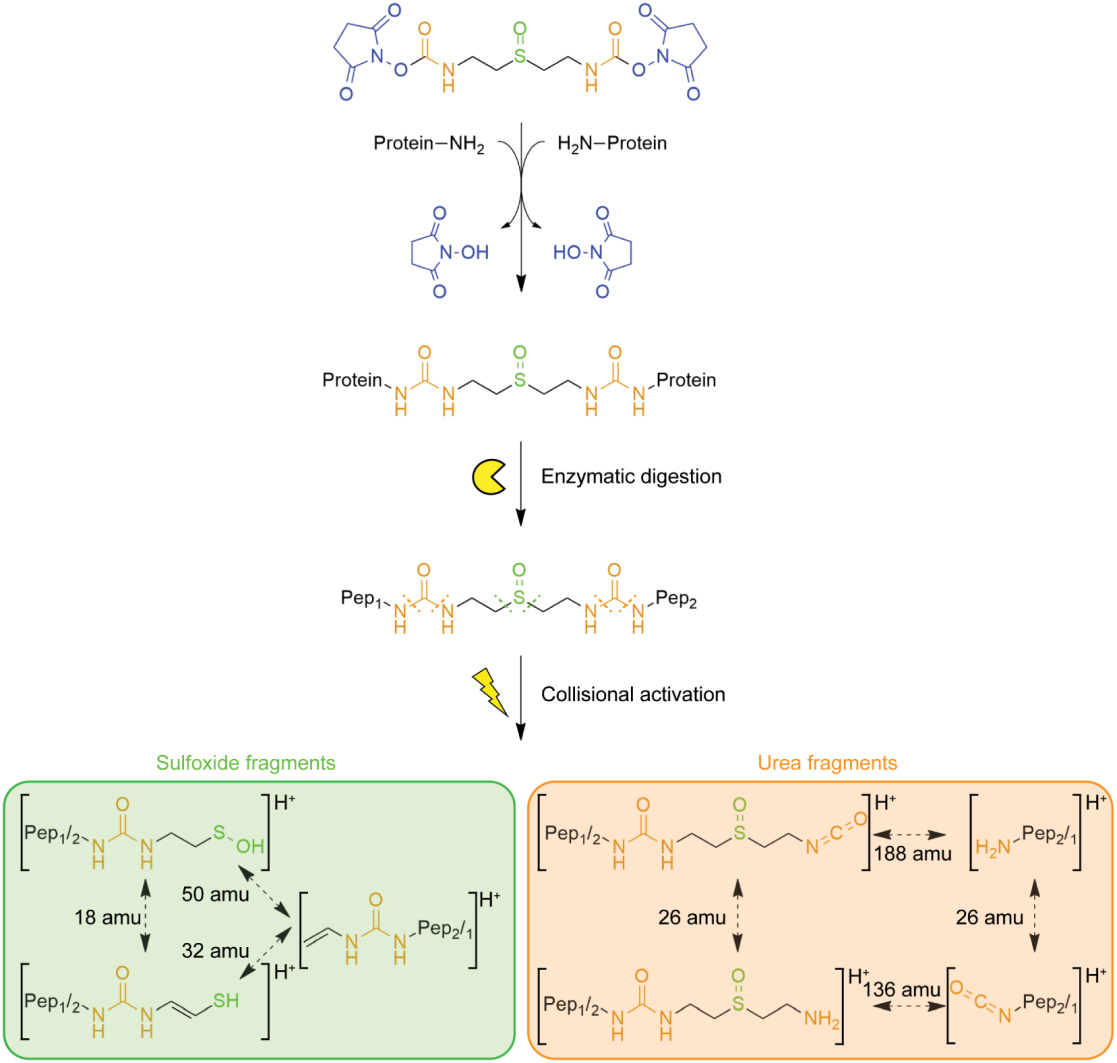
DSSO-carbamate cross-links generate characteristic sulfoxide- and urea-derived fragments upon higher-energy collisional activation. These diagnostic fragments exhibit defined mass shifts and enable the unambiguous identification of cross-linked peptides.

These fragments display the same mass shifts as the DSSO-derived sulfenic, thiol, and alkene species.^3^ In DSSO-carbamate, the two NHS esters of DSSO are replaced by NHS carbamates. This substitution leads to the formation of urea moieties at both ends of the linker upon reaction with protein amine groups. The resulting urea functionalities are well-known MS-cleavable groups, characteristic of urea-based cross-linkers such as DSBU.^2^ Accordingly, we hypothesized that DSSO-carbamate cross-links might also dissociate at the urea sites under HCD conditions (Figure 1). Mono-links, formed when one reactive NHS carbamate group is quenched by water, are expected to contain a carbamic acid moiety. Owing to the low intrinsic stability of carbamic acids in solution, which undergo spontaneous decarboxylation (Figure S2), we included searches for decarboxylated mono-links in our analytical workflow. Additionally, amidated products resulting from mono-link reactions with ammonium bicarbonate (ABC) were also considered. It is worth noting that decarboxylated mono-links are expected to generate only a subset of possible urea-derived fragments upon collisional activation (Figure S2). To experimentally verify the formation of both sulfoxide- and urea-derived fragments, we cross-linked bovine serum albumin (BSA) with DSSO-carbamate and analyzed the tryptic digest using a standard stepped-HCD method. Analysis of the resulting cross-linked BSA peptides with the MeroX software^38,39^ confirmed the presence of all hypothesized sulfoxide- and urea-derived fragment ions under HCD conditions for both cross-links and dead-ends (Figures S3 and S4).

Given the complexity of the gas-phase chemistry and the large variety of possible fragments, we optimized the collision energy conditions for DSSO-carbamate cross-links. Specifically, BSA was cross-linked with DSSO-carbamate for 1 h at room temperature (RT) and at 37 °C. Cross-linked samples were subsequently digested with trypsin and analyzed by LC–MS/MS using stepped-HCD collision energies of 18–21–24, 21–24–27, 24–27–30, and 27–30–33 NCE%. For both RT and 37 °C reactions, the highest number of unique cross-links was identified at stepped-HCD ranges of 24–27–30 and 27–30–33 NCE% (Figure 2A).

**Figure 2:**
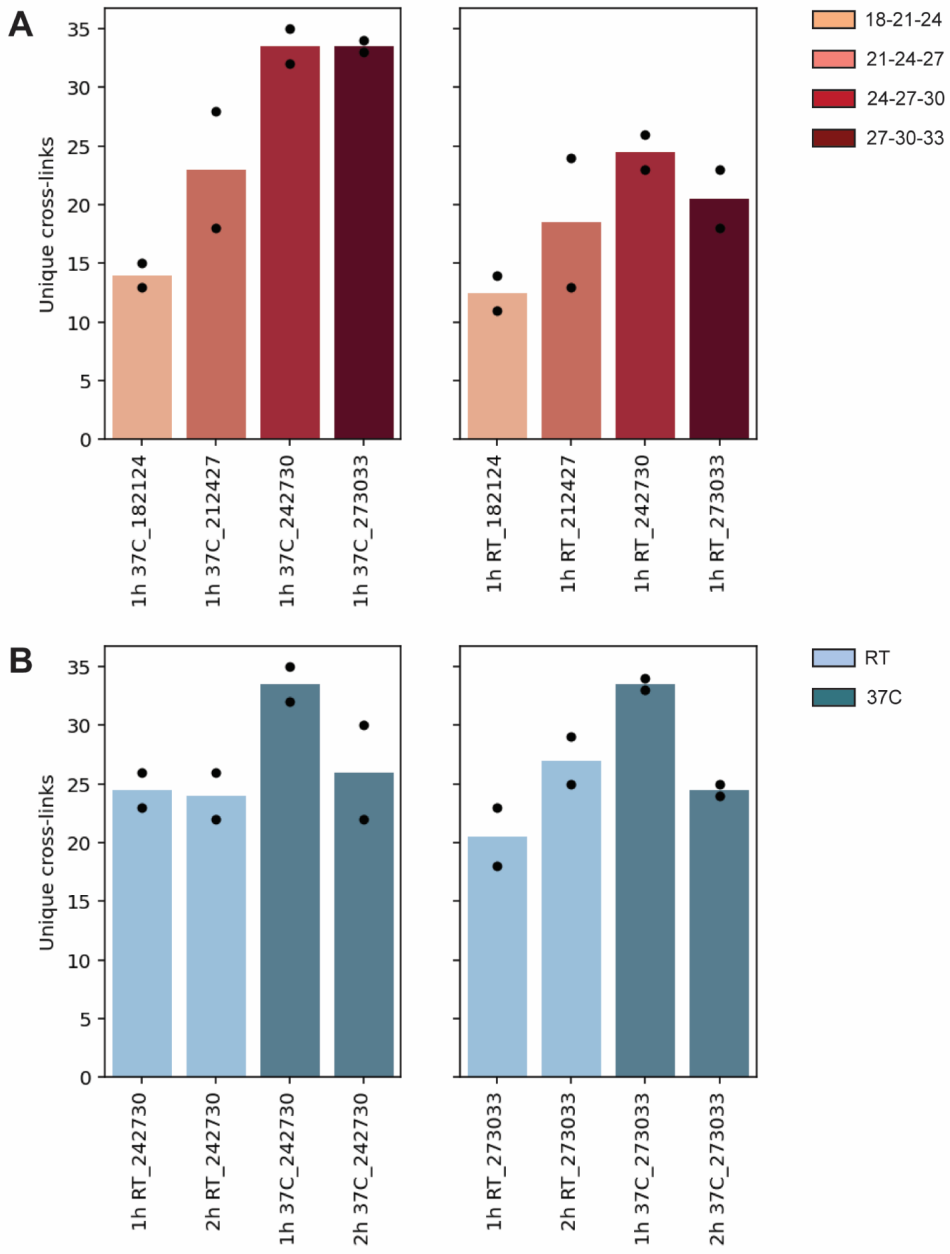
Optimization of collision energy conditions to maximize DSSO-carbamate cross-links identification. (A) Number of unique cross-links identified in BSA after cross-linking at 37 °C or room temperature (RT) for 1 h. Samples were analyzed by LC–MS/MS using different stepped-HCD settings (18–21–24, 21–24–27, 24–27–30, and 27–30–33 NCE%), color-coded from light orange to brown. (B) Number of unique cross-links identified in BSA after cross-linking at 37 °C or RT for 1 and 2 h using the optimized 24–27–30 and 27–30–33 NCE% stepped-HCD regimes. Light blue: RT reactions; dark blue: 37 °C reactions.

These optimized collision energies markedly improved DSSO-carbamate cross-link identification, as higher HCD energies promote the concurrent formation of both sulfoxide and urea fragments. Moreover, increased energy deposition generates a larger number of b- and y-type peptide fragments, resulting in improved sequence coverage for cross-linked peptides. Therefore, we employed these two optimized stepped-HCD methods to determine the optimal cross-linking conditions for DSSO-carbamate in XL-MS experiments.

### In-Solution Reactivity Optimization of DSSO-Carbamate

We evaluated two incubation times, 1 and 2 h, at both room temperature and 37 °C. The resulting four conditions were analyzed in technical duplicate using the optimized stepped-HCD regimes of 24–27–30 and 27–30–33 NCE%. Among all tested conditions, cross-linking at 37 °C for 1 h produced the highest number of unique cross-links (Figure 2B). Doubling the reaction time led to a slight increase in the number of identified cross-links at RT under the 27–30–33 stepped-HCD regime, whereas a small decrease was observed at 37 °C for both collision energy ranges.

### Site Reactivity Analysis of DSSO-Carbamate Cross-Links

We next analyzed the site distribution of cross-links in BSA after DSSO-carbamate treatment. Accurate cross-link site assignment critically depends on the detection of diagnostic b and y fragment ions bearing cross-linker modifications, which in turn are influenced by the applied collision energy. Unexpectedly, we observed a remarkably high fraction of cross-links involving the mature BSA N-terminal peptide (sequence DTHKSEIAHR). The proportion of N-terminal-containing cross-links obtained with the two best-performing stepped-HCD methods averaged between 40% and 50% across the different experimental conditions (Figure 3). For both stepped-HCD regimes used in this study, these values represent a substantial fraction of all BSA cross-links. In most cases, the N-terminus was unambiguously identified as the cross-linking site (Figures S3 and S5A-B). In other instances, although the N-terminally modified b_1_ ion was absent, the y_6_–y_7_–y_9_ ion series excluded the adjacent threonine (T), lysine (K), and serine (S) residues (Figure S5C). This trend appears to be a distinctive feature of DSSO-carbamate, as cross-links involving the BSA N-terminus are typically much less abundant than lysine–lysine linkages.^40^ This observation is particularly striking considering the expected 1:59 ratio of N-terminal to lysine amine groups in BSA. Such a marked deviation strongly suggests an enhanced reactivity of the NHS carbamate moiety toward the protein N-terminal α-amine compared to conventional NHS esters. These findings led us to hypothesize that homobifunctional NHS carbamate cross-linkers may exhibit an intrinsic bias toward N-terminal modification. Consistently, comparison of BSA DSSO-carbamate cross-linking sites with those obtained using the NHS ester DSBU (PXD021648,^40^ Orbitrap-based acquisition) indicates that the two reagents provide complementary structural restraints, despite the higher overall reactivity of DSBU. Notably ∼60% of the 19 cross-links uniquely identified with DSSO-carbamate involved the BSA N-terminus.

**Figure 3:**
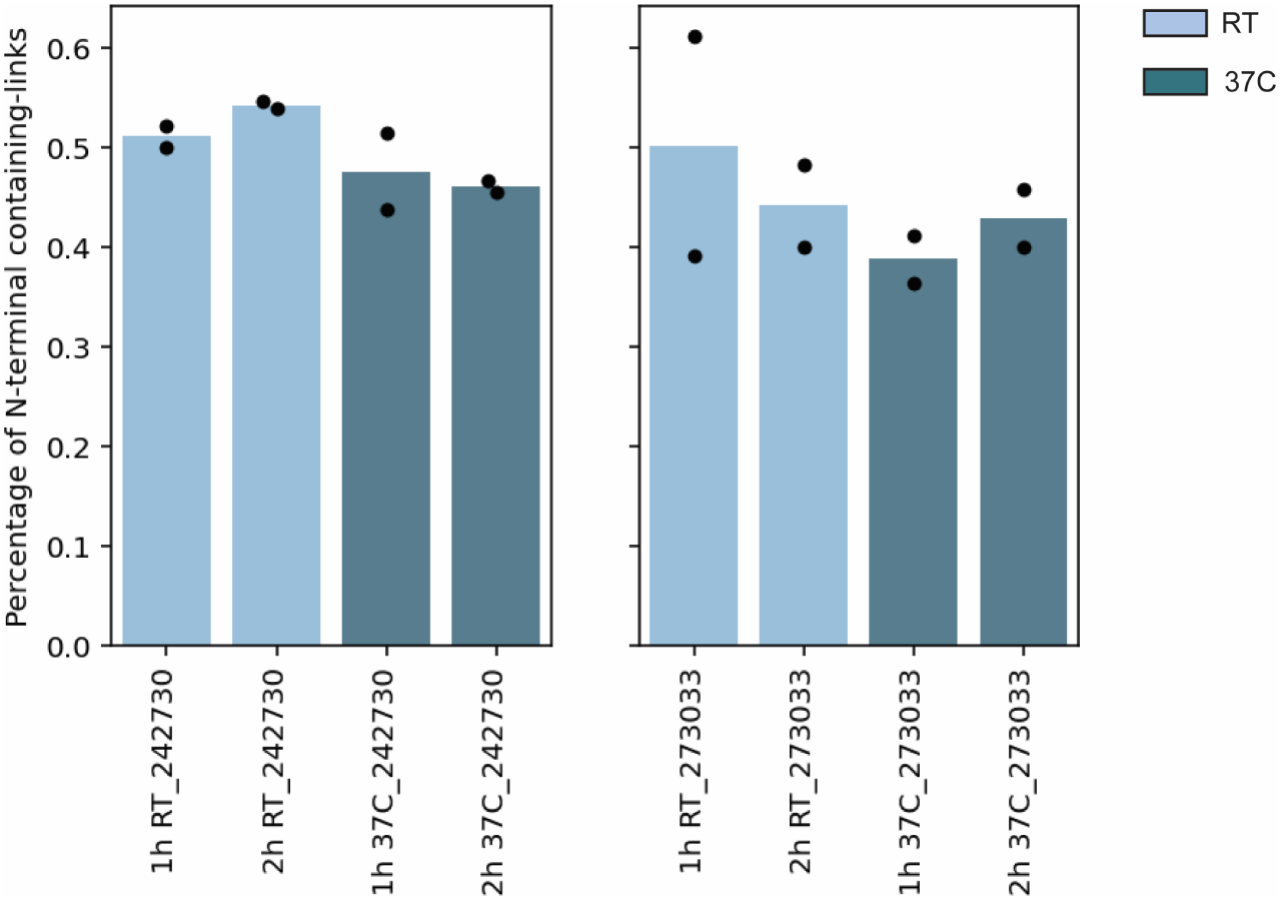
(A) Analysis of reactive sites for DSSO-carbamate cross-links. A high proportion (40–50%) of DSSO-carbamate cross-links involves the N-terminal BSA peptide. Light blue: reactions at room temperature (RT); dark blue: reactions at 37 °C.

### Reaction of DSSO-Carbamate Cross-Linker with N-Terminally Acetylated and Nonacetylated Alpha-Synuclein

To further investigate the N-terminal reactivity of DSSO-carbamate, we performed an additional XL–MS experiment. Specifically, α-synuclein (α-syn) was recombinantly expressed and purified in both its unmodified and N-terminally acetylated forms (NAc_α-syn).^37^ Both proteins are intrinsically disordered and therefore highly reactive toward chemical cross-linkers.^41^ Cross-linking reactions were carried out using low (2-fold and 10-fold) molar excesses of either DSSO-carbamate or the NHS ester–based cross-linker DSBU, followed by analysis with native mass spectrometry (Figures S6 and S7). For DSBU, the average number of modifications decreased modestly from 5 to 4 (2-fold) and from 9 to 8 (10-fold) upon N-terminal acetylation of α-syn (Figure 4 and S6). Some unreacted protein was still detectable at 2-fold excess, whereas at 10-fold excess all protein species were modified. In contrast, for DSSO-carbamate, the average number of modifications dropped more sharply, from 2 to 0 (2-fold) and from 5 to 2 (10-fold), upon N-terminal acetylation (Figure 5). In both cases, unreacted α-syn represented the predominant species even at 10-fold DSSO-carbamate excess. Beyond the inherently lower reactivity of DSSO-carbamate compared to the NHS ester–based DSBU, N-terminal acetylation led to an almost complete suppression of protein cross-linking by DSSO-carbamate, whereas DSBU reactivity was only slightly affected.

**Figure 4:**
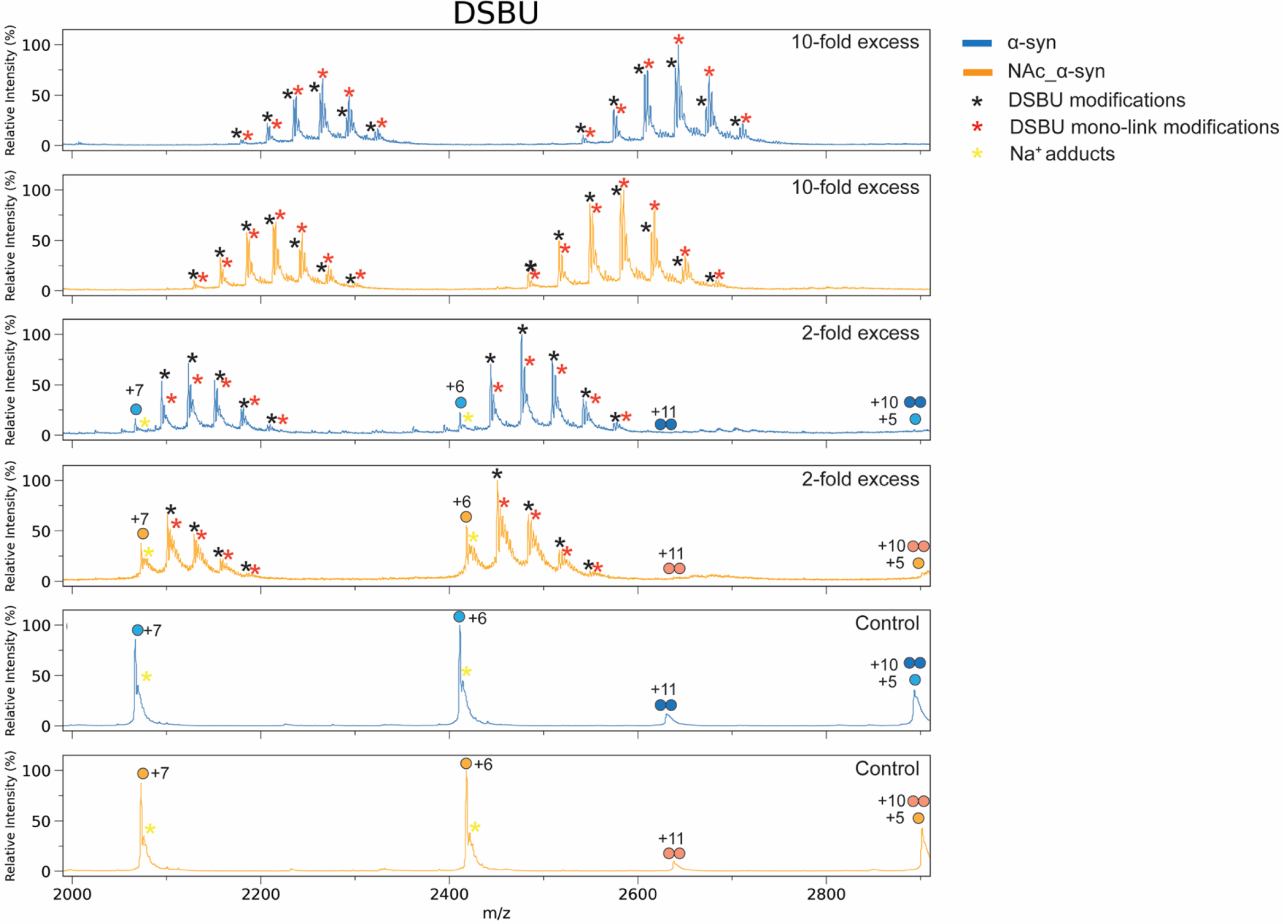
Native mass spectra of DSBU-cross-linked α-syn (blue) and NAc_α-syn (orange). Control (no DSBU), 2-fold, and 10-fold DSBU excess conditions are shown and overlaid for comparison.

**Figure 5:**
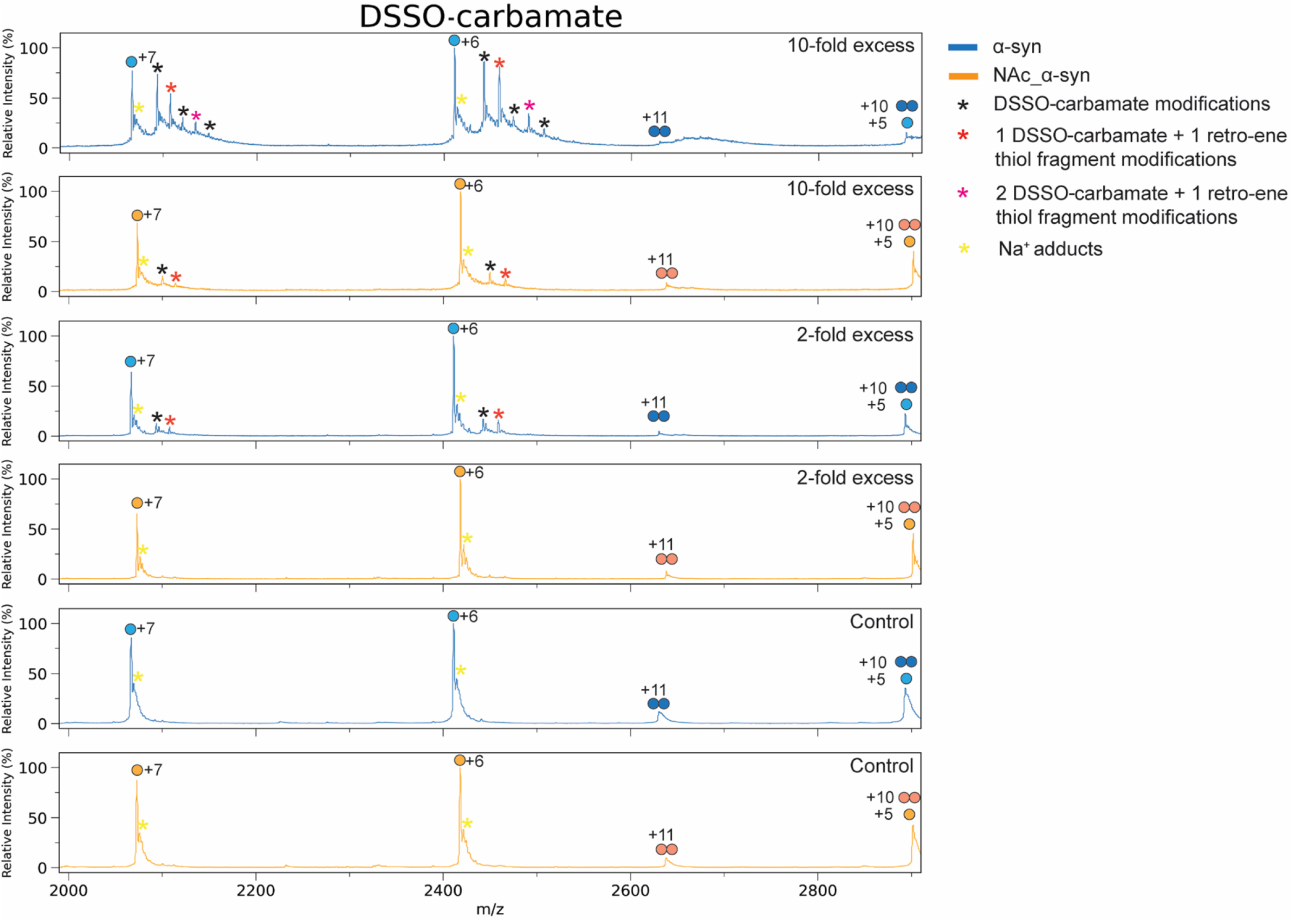
Native mass spectra of DSSO-carbamate-cross-linked α-syn (blue) and NAc_α-syn (orange). Control (no DSSO-carbamate), 2-fold, and 10-fold DSSO-carbamate excess conditions are shown and overlaid for comparison.

If the number of cross-linked species in the α-syn sample is considered as the sum of N-terminally linked species (e.g., Nterm–K) and lysine–lysine (K–K) species, in NAc_α-syn this contribution originates exclusively from K–K linkages, as the acetylated N-terminus cannot react with the cross-linker. We therefore calculated a modification penalty rate (Δ_acetyl_) for both reagents. Specifically, the summed intensities of cross-linked species for α-syn and NAc_α-syn were divided by the total intensity of all species (including the unreacted protein) within the most abundant 6+ charge state signal. The 10-fold DSSO-carbamate and 2-fold DSBU conditions were used for this analysis, as these still contained a measurable amount of unreacted protein.

The 10-fold DSBU condition was excluded because it resulted in over-modification of the proteins. The difference between the modification rates for the nonacetylated and acetylated forms (nonacetylated–acetylated) reflects the penalty due to N-terminal acetylation. For DSBU, Δ_acetyl_ was 13.8% (decreasing from 92.8% to 79.0% modification rate), whereas for DSSO-carbamate, Δ_acetyl_ increased to 41.3% (decreasing from 57.9% to 16.6%), confirming a pronounced preference of NHS carbamates to react with the protein N-terminus. Taken together, our bottom-up analysis and intact-protein experiments consistently demonstrate that homobifunctional NHS carbamate cross-linkers display a marked tendency to react with N-termini compared to conventional NHS esters. Although less reactive overall, NHS carbamates yield complementary structural information and are particularly useful for probing protein complexes in which N-terminal interactions play a critical role.

### Reaction of NNP9 Cross-Linker with Infliximab-TNFα

To generalize the observed N-terminal selectivity of DSSO-carbamate to other NHS carbamate–based reagents, the NNP9 cross-linker was selected as an alternative probe. Unlike DSSO-carbamate, NNP9 contains a rigid phenyl core with a maximum spacer length of 10 Å, but both reagents share the same two NHS carbamate reactive head groups. The Infliximab–TNFα complex had previously been characterized using the NHS ester-based DSBU cross-linker.^42^ DSBU cross-linking revealed the essential role of the N-terminus of the Infliximab light chain (LC) in target binding. Several cross-links were detected between the complementarity-determining regions (CDRs) of the LC and heavy chain (HC) of Infliximab and the C–D and E–F loops of TNFα. Additionally, DSBU identified a cross-link between the N-terminal aspartic acid of framework region 1 of the LC and multiple residues within the C–D and E–F loops of TNFα. In contrast, the HC N-terminus did not interact with TNFα, suggesting a specific interaction of the LC N-terminus with the receptor. When NNP9 was employed (Figure S8), cross-linking was observed exclusively between the LC N-terminal region and the C–D loop of TNFα, indicating a strong preference for reaction with the N-terminal paratope of Infliximab. The relative percentages of N-terminal–containing links among the total cross-linked spectrum matches (CSMs) and unique cross-links for NNP9 and DSBU were calculated and compared. NNP9 showed a markedly higher proportion of N-terminal-containing links than DSBU (58.1% vs 12.1% for CSMs; 30.3% vs 9.4% for unique cross-links, Figure 6). The intensity distribution of N-terminal-containing (N term–K/S/T/Y) versus lysine-containing (K–K/S/T/Y) cross-links additionally supports the higher propensity of NHS carbamate-based reagents to target protein N-termini (Figure S9). Overall, the results obtained for NNP9 are consistent with those observed for DSSO-carbamate and further support the notion that NHS carbamate-based cross-linkers exhibit enhanced selectivity toward protein N-termini, providing complementary structural insights in protein–protein interaction studies.

**Figure 6:**
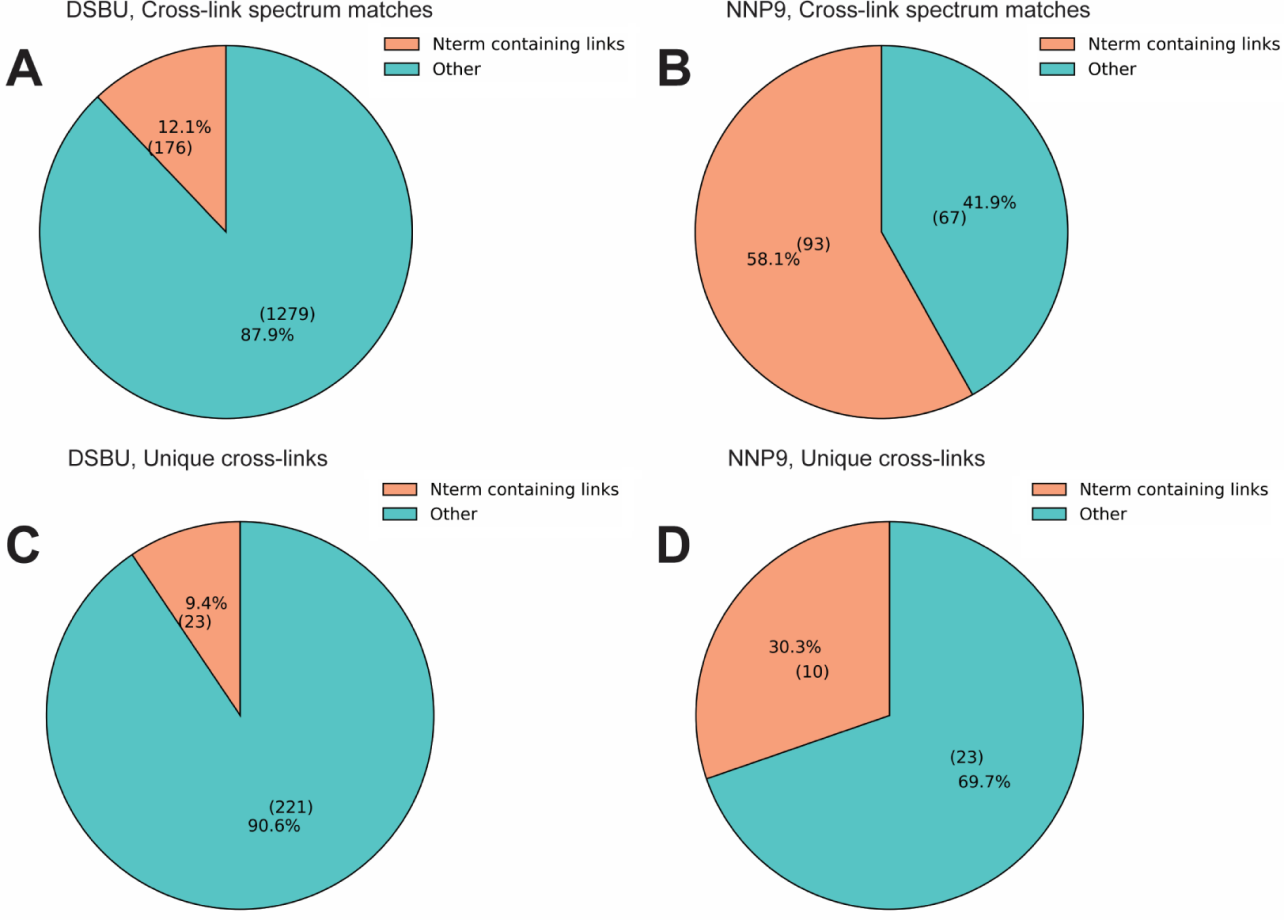
Relative percentages of cross-links involving the N-terminus compared to the total number of cross-link spectrum matches (CSMs) (A, DSBU; B, NNP9) and unique cross-links (C, DSBU; D, NNP9). Results represent the combined data from LC–MS acquisitions performed at 50-fold and 100-fold cross-linker excess.

### Evaluation of NHS Carbamate-Based Reagents for Complex Samples

To further benchmark the behavior of NHS carbamate chemistry in complex samples, we performed a proteome-wide comparison between an NHS carbamate-based cross-linker (NNP9) and an NHS ester-based reagent (DSBU). These two cross-linkers were respectively employed to study the interactomes of *Neisseria meningitidis* (serogroup C strain 8013)^43^ and *Drosophila melanogaster*.^39^ Because N-terminal processing can lead to missing or misassigned identifications, cross-link searches were conducted against a combined database containing already mature proteins along with protein sequences generated by removing UniProt-annotated signal peptides and propeptides. This “dual-fasta” strategy increased sensitivity toward N-terminus-containing cross-links, which could be underrepresented in proteome-wide workflows relying exclusively on full-length sequences. Importantly, for the NNP9 data set, also the MS-cleavable fragments generated after click-chemistry enrichment were considered (Figure S10A,B). Notably, the NHS-carbamate NNP9 data set yielded a markedly higher fraction of cross-links involving protein N-termini (>20% for all three biological replicates) compared to the NHS ester DSBU data set (<5% for all the different SEC fractions), supporting the preferential N-terminal reactivity of this chemistry and highlighting its complementarity to conventional NHS ester reagents (Figure 7A). This enrichment in N-terminal restraints is confirmed by the low number of N-termini sites compared to the whole number of cross-linkable sites (only lysine residues for simplicity) contained in each model organism proteome (Figure S11). Within all the NNP9 identified cross-links, a very high percentage (>85%) was coming from homo-oligomeric complexes (Figure 7B).

**Figure 7:**
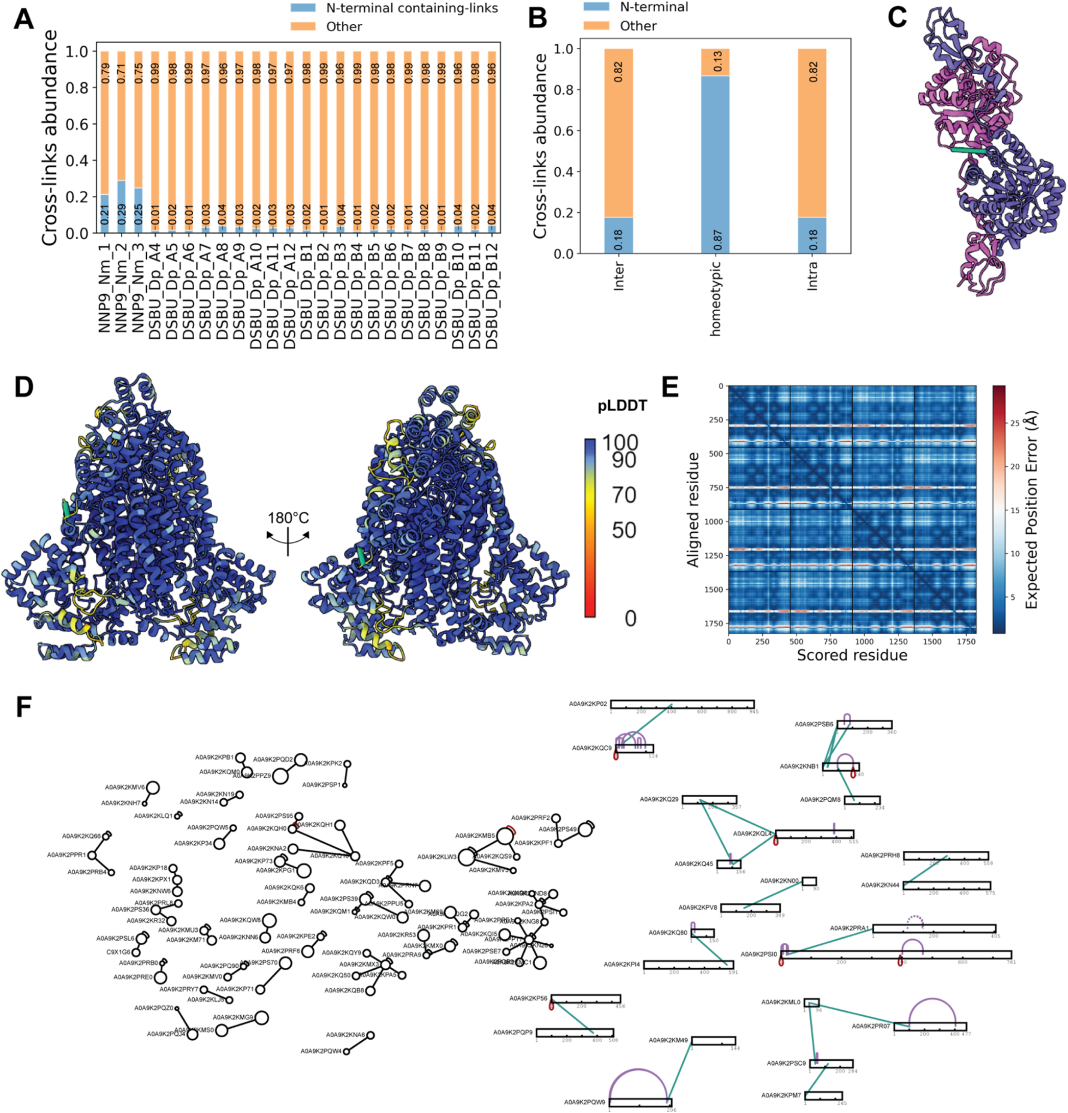
(A) Fraction of N-terminal containing cross-links for the three biological replicates of the NHS carbamate NNP9 data set on *Neisseria meningitidis* and for all the SEC fractions of the NHS ester DSBU data set on *Drosophila melanogaster*. The percentage of N-terminal restraints is always higher than 20% for all the NNP9 runs, while it ranges between 1 and 4% for all the DSBU runs. (B) Fraction of N-terminal inter-, homeotypic and intramolecular restraints for the NNP9 data set. (C) NNP9 homeotypic cross-links confirm the head-to-head homodimeric structure of siaC (pdb id: 1XUU). (D) NNP9 homeotypic cross-links validate the homotetrameric architecture of the AlphaFold3 predicted purB. (E) Predicted aligned error plot for the homotetramer purB predicted by AlphaFold3, showing a very high confidence of the prediction. (F) Exemplified interaction map obtained with the NNP9 cross-linker. On the right side, N-terminal interactions are displayed as bar plots.

From a structural perspective, the enhanced detection of these homeotypic N-terminally derived restraints is particularly informative for assemblies in which N-termini are in close spatial proximity, such as head-to-head dimers or higher-order oligomers. This was exemplified in the case of sialic acid synthase (NeuB/SiaC, pdb id: 1XUU^44^), where an identified NNP9 homeotypic link recapitulated the X-ray head-to-head SiaC dimer (Figure 7C). Additionally, the homotetrameric adenylosuccinate lyase purB, homologous to the solved structures from *E. coli* and other gram-negative bacteria,^45^ was predicted with high confidence by AlphaFold3 (Figure 7D,E), and its head-to-head dimer of dimer architecture confirmed by NNP9 cross-links. An interaction map for the NNP9 data set is shown in Figure 7F, where ∼20% of inter/intramolecular cross-links were identified within protein N-termini.

## Conclusions and Outlook

In this work, we unveiled the complex gas-phase chemistry of the previously developed cross-linker DSSO-carbamate. The replacement of the classical NHS ester warheads with two NHS carbamates leads to the formation of urea-type MS-cleavable groups on each side of the molecule, which profoundly influences its overall fragmentation pattern. In XL–MS analyses, sulfoxide- and urea-derived fragments are typically generated upon collisional activation, such as higher-energy collisional dissociation (HCD). Accounting for all these possible fragment types during data processing significantly enhances cross-link identification rates.

We optimized both the collision energy settings and the in-solution reactivity of DSSO-carbamate to improve its analytical performance. Stepped-HCD methods employing higher normalized collision energy (NCE) ranges, specifically 24–27–30 and 27–30–33 NCE%, yielded the highest numbers of unique cross-links in the model protein BSA. Regarding reaction conditions, cross-linking for 1 h at 37 °C provided the best results under protein-compatible buffer conditions.

Unexpectedly, a large fraction of N-terminal-containing cross-links was observed, indicating enhanced reactivity of DSSO-carbamate toward protein N-termini compared to traditional NHS ester-based reagents. To further investigate this phenomenon, we performed intact-protein cross-linking experiments using α-synuclein (α-syn) and its N-terminally acetylated form (NAc_α-syn). These experiments confirmed the preferential reactivity of DSSO-carbamate for the free protein N-terminus, a feature that may be particularly advantageous for studying protein complexes in which N-terminal interactions are functionally relevant.

Finally, we demonstrated that this N-terminal selectivity is not unique to DSSO-carbamate but extends to other NHS carbamate-based reagents, such as NNP9. Using the Infliximab–TNFα complex as a model system, where the N-terminal region of the light variable chain plays a critical role in target binding, we observed a comparable N-terminal bias for NNP9 relative to DSBU. Although NHS carbamate-based cross-linkers display lower overall reactivity than their NHS ester counterparts, this limitation can be mitigated by increasing cross-linker concentration. Finally, we also highlighted the complementarity of NHS carbamate-based cross-linkers in proteome-wide XL-MS studies. The NHS carbamate warhead is well established in covalent drug design and chemoproteomics.^46,47^ Therefore, beyond XL–MS applications, our findings highlight the broader relevance of NHS carbamate chemistry for the development of targeted bioconjugation strategies and chemoproteomic probes.

## Supplementary Material







## Notes

MeroX data analysis settings and FASTA files for all the different data sets; additional information regarding DSSO carbamate molecular properties (Figure S1), gas-phase behavior of DSSO-carbamate mono-links (Figure S2), fragment ion spectra of DSSO-carbamate cross-links and mono-links (Figures S3–S5), full native mass spectra of α-syn and NAc_α-syn (Figures S6-S7), SDS-PAGE for Infliximab-TNFα cross-linked with NNP9 (Figure S8), intensities of Infliximab-TNFα DSBU and NNP9 cross-links (Figure S9), gas-phase behavior of NNP9 cross-links (Figure S10), and fraction of N-terminal sites of the investigated proteomes (Figure S11) (PDF)
MeroX data analysis settings for all the different data sets (TXT)
FASTA files for all the different data sets (TXT)

## Notes

The mass spectrometry proteomics data have been deposited to the ProteomeXchange Consortium via the PRIDE partner repository with the dataset identifier PXD069252.
