# Supplementary material for: Intrinsic N‑Terminal Reactivity and Improved Analysis of DSSO-Carbamate and Carbamate-Based Cross-Linkers
Source: Anal Chem. 2026 Mar 11;98(11):8167–78. doi: 10.1021/acs.analchem.5c06834 (PMC13019429; doi:10.1021/acs.analchem.5c06834)
Supplement: Supplementary file 1 [file ac5c06834_si_001.pdf]

Supporting Information

Intrinsic N-Terminal Reactivity and Improved Analysis of DSSO-Carbamate and Carbamate-based Cross-linkers

Alessio Di Ianni*[a,b,c], Thomas Fabian Leischner[d], Bogdan-Razvan Brutiu[d], Iakovos Saridakis[d], Andrea Di Ianni[e,f], Hendrik Krolle[a,b,g,h,i], Christian H. Ihling[a,b], Saad Shaaban[d], Nuno Maulide*[d], Andrea Sinz*[a,b], and Claudio Iacobucci*[l]

[a] Center for Structural Mass Spectrometry, Martin Luther University Halle-Wittenberg, Kurt-Mothes-Str. 3, D-06120 Halle/Saale, Germany

[b] Department of Pharmaceutical Chemistry & Bioanalytics, Institute of Pharmacy, Martin Luther University Halle-Wittenberg, Kurt-Mothes-Str. 3, D-06120 Halle/Saale, Germany

[c] Human Technopole, V.le Rita Levi Montalcini 1, Milan, 20157, Italy

[d] Institute of Organic Chemistry, University of Vienna, 1090 Wien, Austria

[e] University of Turin, Molecular Biotechnology Center, Department of Molecular Biotechnology and Health Sciences, University of Turin, Turin 10126, Italy

[f] NBE-DMPK Innovative BioAnalytics, Merck Serono RBM S.p.A., an Affiliate of Merck KGaA, Darmstadt, Germany, Via Ribes 1, Colleretto Giacosa (TO) 10010, Italy

[g] MS Vision, Televisieweg 40, 1322 AW Almere, The Netherlands

[h] Division of Bioanalytical Chemistry, Vrije Universiteit Amsterdam, De Boelelaan 1105, 1081 HV Amsterdam, The Netherlands

[i] Centre for Analytical Sciences Amsterdam, 1098 XH Amsterdam, The Netherlands

[l] Department of Physical and Chemical Sciences, University of L'Aquila, Via Vetoio, L'Aquila, 67100, Italy

Email: alessio.diianni@fht.org, claudio.iacobucci@univaq.it, andrea.sinz@pharmazie.uni-halle.de, nuno.maulide@univie.ac.at

Table of Contents

Data analysis settings..3

Supplementary Figures 5

References...14

Data analysis settings

Analysis of DSSO-carbamate *BSA cross-links*

Identification of cross-links was performed with MeroX 2.0.1.7 [1-2]. The following settings were applied: Proteolytic cleavage C-terminally at Lys and Arg (up to 3 missed cleavages were allowed); peptide length: 4 to 30 amino acids; modifications: alkylation of Cys by iodoacetamide (fixed), oxidation of Met (variable); cross-linker specificity: Lys, Ser, Thr, Tyr, N-terminus; search algorithm: RISEUP with 3 max missing ions; precursor mass accuracy: 10 ppm; fragment ion mass accuracy: 20 ppm; signal-to-noise ratio > 2; precursor mass correction enabled; false discovery rate (FDR) cut-off: 1%, and minimum score cut-off: 20.

DSSO-carbamate cross-linker mass modification: $C_6H_8N_2O_3S$ (188.026 amu);

DSSO-carbamate sulfoxide fragments: A – C_3H_3NO (69.022 amu), T – C_3H_3NOS (100.994 amu), S – $C_3H_5NO_2S$ (119.004 amu);

DSSO-carbamate urea fragments: Pep-DSSO-NCO – $C_6H_8N_2O_3S$ (188.026 amu), Pep-DSSO-NH_2 – $C_5H_{10}N_2O_2S$ (162.046 amu), Pep-NCO – CO-H_2 (25.979 amu);

DSSO-carbamate reporter ions: 206_reporter – $C_6H_{12}N_3O_3S$ (206.060 amu), 180_reporter – $C_5H_{14}N_3O_2S$ (180.081 amu), 163_reporter – $C_5H_{11}N_2O_2S$ (163.054 amu), 137_reporter – $C_4H_{13}N_2OS$ (137.075 amu)

Analysis of NNP9 Infliximab-*TNFα cross-links*

Identification of cross-links was performed with MeroX 2.0.1.4. The following settings were applied: Proteolytic cleavage C-terminally at Lys and Arg and C-terminally at Met, Phe, Leu, Trp and Tyr (up to 3 missed cleavages were allowed); peptide length: 5 to 30 amino acids; modifications: alkylation of Cys by iodoacetamide (fixed), oxidation of Met (variable); cross-linker specificity: Lys, Ser, Thr, Tyr, N-terminus; search algorithm: Quadratic mode; precursor mass accuracy: 10 ppm; fragment ion mass accuracy: 20 ppm; signal-to-noise ratio > 2; precursor mass correction enabled; applied prescore intensity:10%; false discovery rate (FDR) cut-off: 1%, and minimum score cut-off: 50.

NNP9 cross-linker mass modification: $C_{14}H_{14}N_6O_3$ (314.113 amu);

NNP9 fragments: Pep_NCO – CO-H_2 (25.979 amu), Pep_NNP9_NH_2 – $C_{13}H_{16}N_6O_2$ (288.133 amu), Pep_NNP9_NCO – $C_{14}H_{14}N_6O_3$ (314.113 amu)

Analysis of DSBU Infliximab-*TNFα cross-links*

Identification of cross-links was performed using MeroX 2.0.1.4. Settings applied for data analysis were taken from Di Ianni et al[3].

Analysis of NNP9 Neisseria Meningitidis proteome-wide dataset

Identification of cross-links was performed with MeroX 2.0.1.7. The following settings were applied: Proteolytic cleavage C-terminally at Lys and Arg (up to 4 missed cleavages were allowed); peptide length: 5 to 30 amino acids; modifications: alkylation of Cys by iodoacetamide (fixed), oxidation of Met (variable); cross-linker specificity: Lys, Ser, Thr, Tyr, N-terminus; search algorithm: proteome-wide mode with minimum peptide score 10 ; precursor mass accuracy: 10 ppm; fragment ion mass accuracy: 20 ppm; signal-to-noise ratio > 2; precursor mass correction disabled; false discovery rate (FDR) cut-off: 1%, and minimum score cut-off: 20.

NNP9-PC alkyne cross-linker mass modification: $C_{17}H_{19}N_7O_3$ (369.155 amu);

NNP9-PC alkyne fragments: Pep_NCO – CO-H_2 (25.979 amu), Pep_NNP9-PCalkyne_NH_2 – $C_{16}H_{21}N_7O_2$ (343.176 amu), Pep_NNP9_NCO – $C_{17}H_{19}N_7O_3$ (369.155 amu)

NNP9-PC alkyne main reporter ions: 370_reporter – $C_{17}H_{20}N_7O_3^+$ (370.162 amu), 344_reporter – $C_{16}H_{22}N_6O_2^+$ (344.183 amu), 318_reporter – $C_{15}H_{24}N_7O^+$ (318.204 amu)

Analysis of DSBU Drosophila Melanogaster proteome-wide dataset

Identification of cross-links was performed with MeroX 2.0.1.7. Settings applied for data analysis were taken from Götze et al [2].

Data visualization

Plots were generated using the *matplotlib* and *seaborn* python packages.

Supplementary Figures

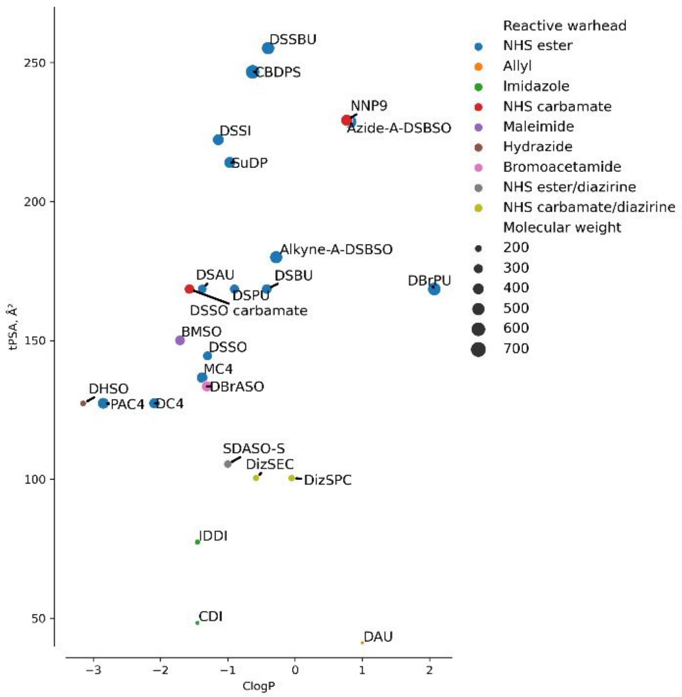

Figure S1. Molecular properties of DSSO-carbamate and other MS-cleavable cross-linkers. Plotted is the Topological Polar Surface Area (tPSA, Å) vs computed partition coefficient (ClogP) for the main MS-cleavable cross-linkers.

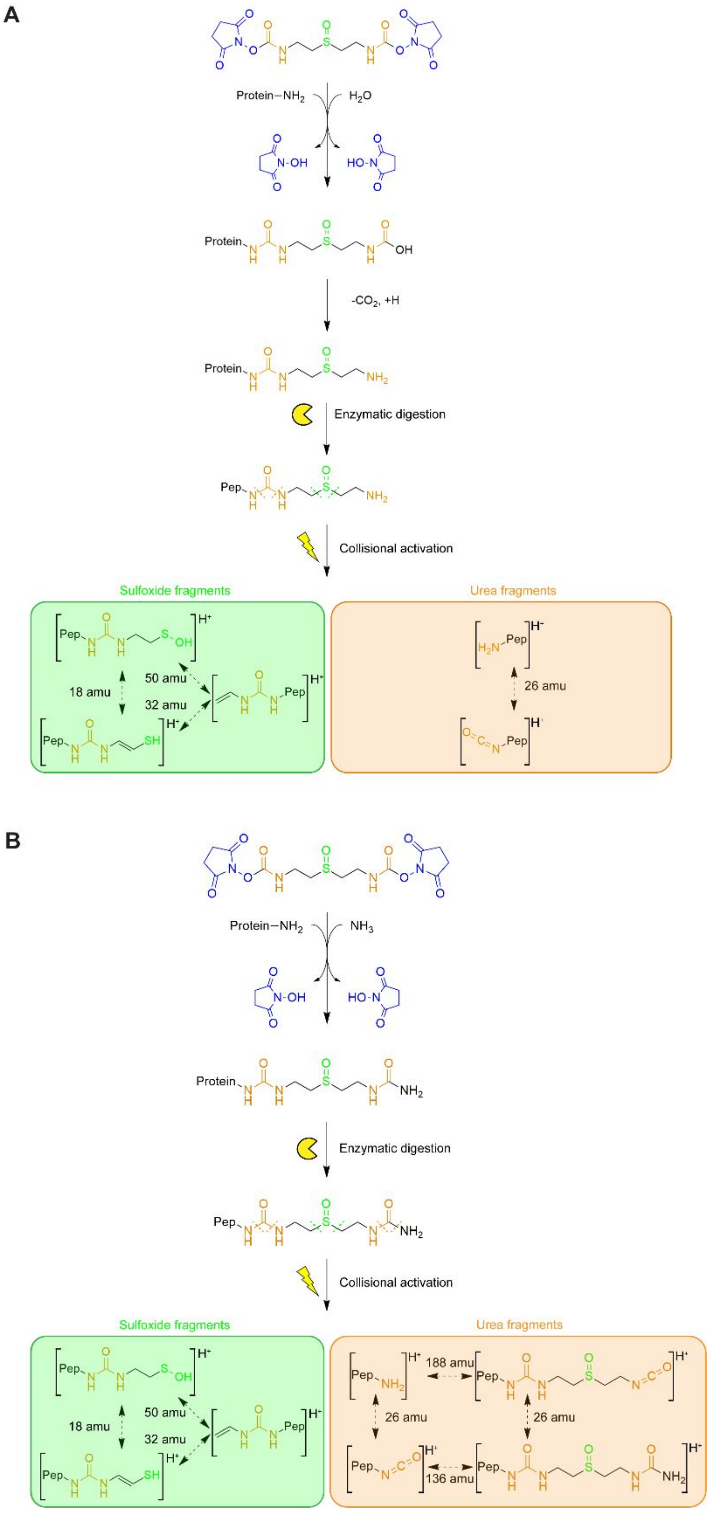

Figure S2. DSSO-carbamate gas-phase dissociation behavior under collisional activation conditions for mono-links. Two types of DSSO-carbamate mono-links can be generated after XL-MS reaction. (A) After water hydrolysis, the resulting carbamic acid product quickly decarboxylates in solution. (B) After ammonia quenching, a resulting amidated DSSO-carbamate mono-link is also generated. These two types of mono-links generate different subsets of urea fragments upon collisional activation.

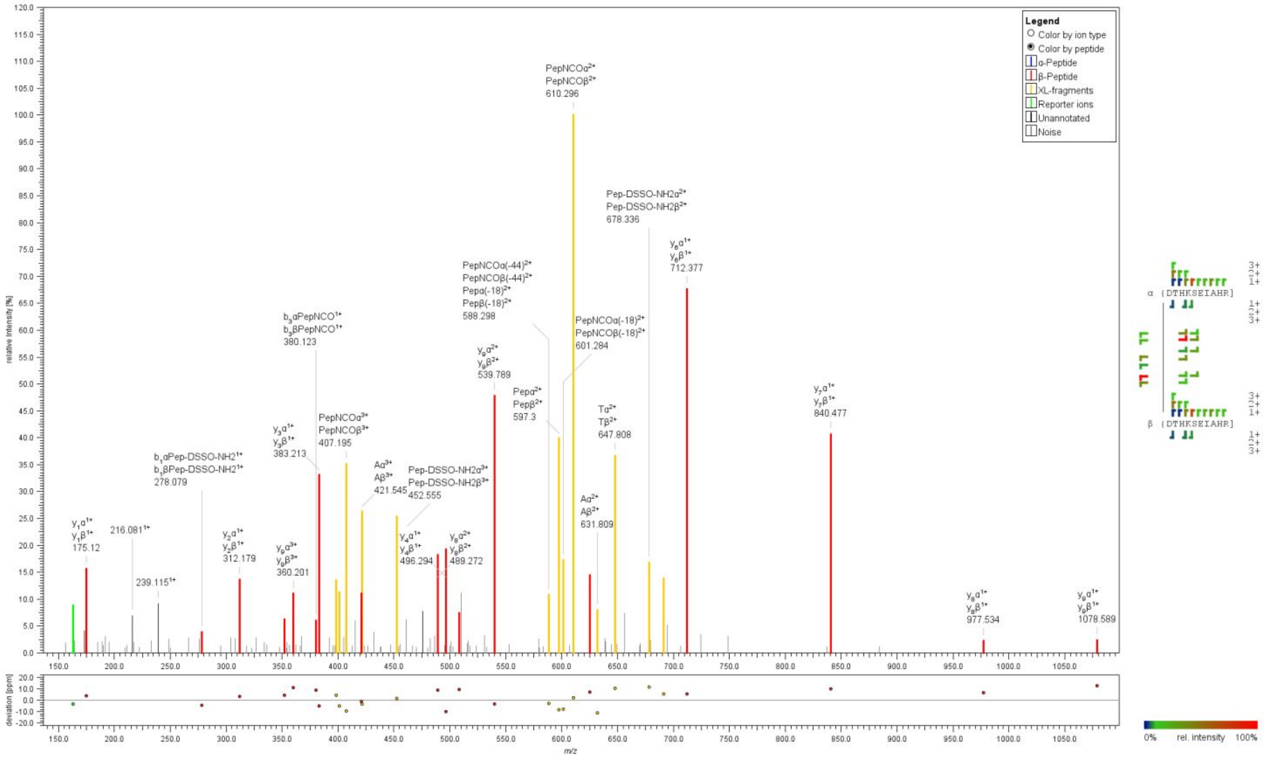

Figure S3. Exemplary fragment ion spectrum of a DSSO-carbamate BSA cross-link under higher-energy collisional dissociation. MS-cleavable fragments are displayed in yellow, b/y ion fragments are colored in red, reporter ions are indicated in green.

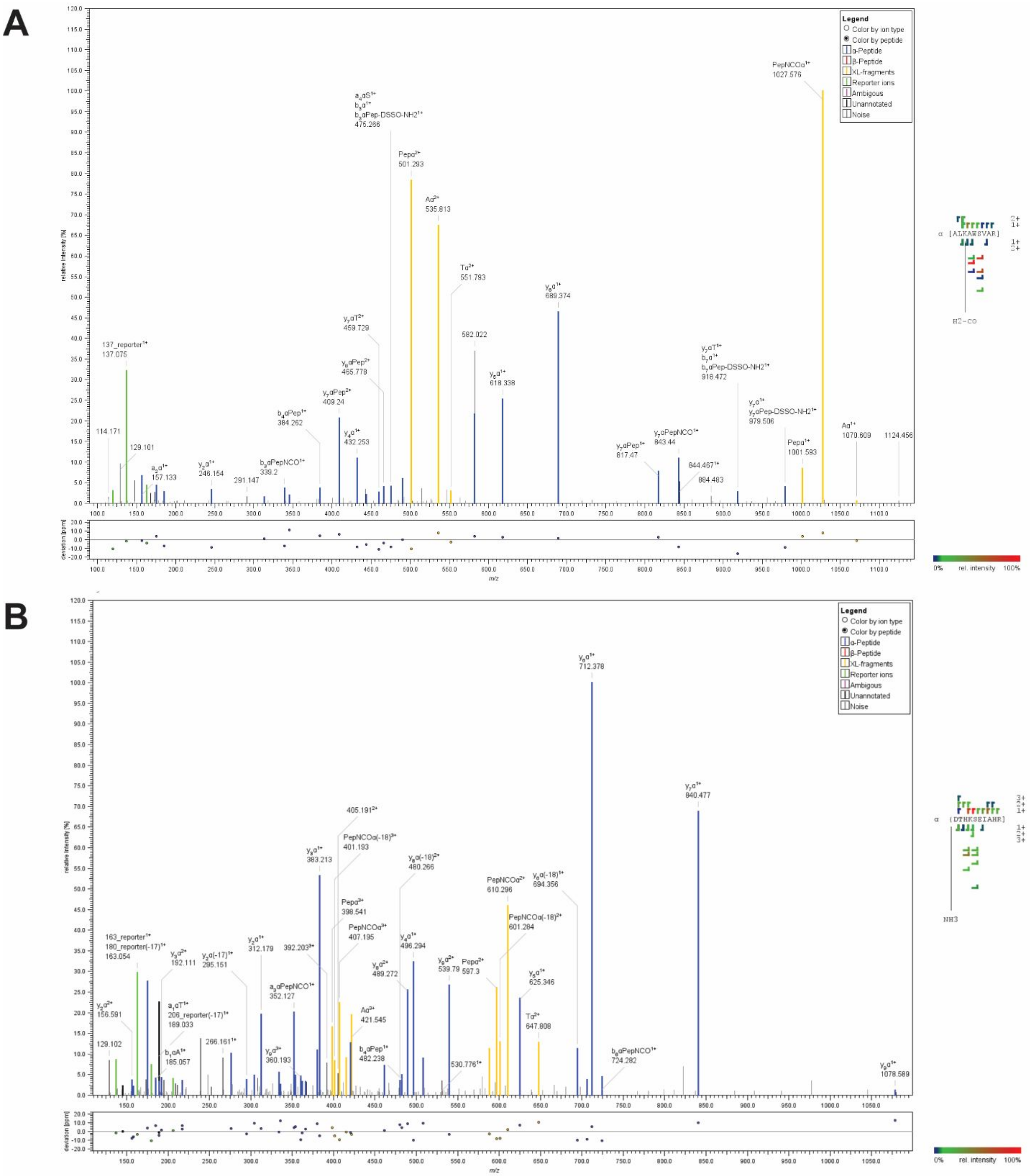

Figure S4. Exemplary fragment ion spectra of two different DSSO-carbamate BSA mono-links under higher-energy collisional dissociation. MS-cleavable fragments are displayed in yellow, b/y ion fragments are colored in blue, reporter ions are indicated in green.

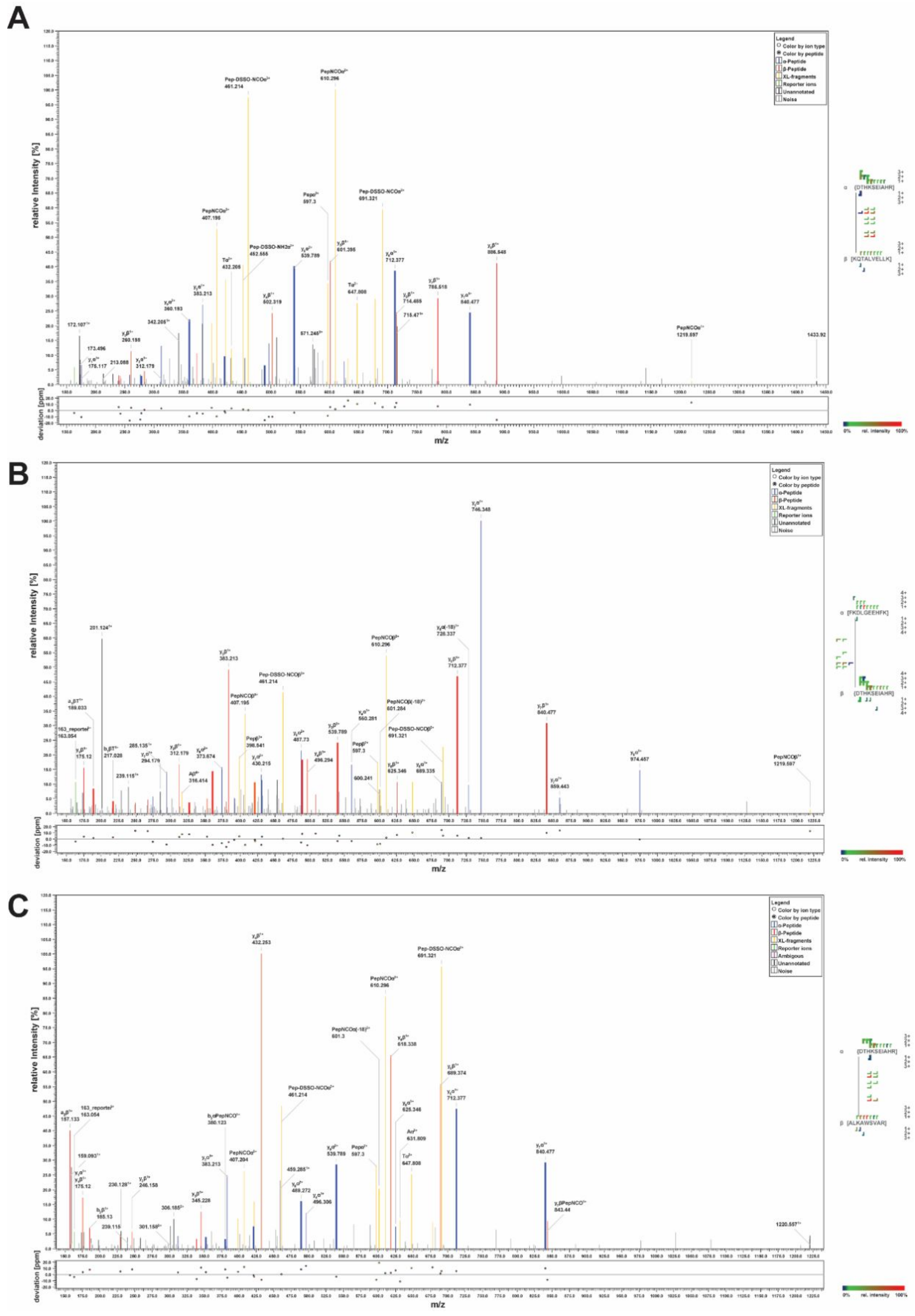
A
B
C
relative intensity [%]
deviation [ppm]
m/z
Legend
Color by ion type
Color by peptide
α-Peptide
β-Peptide
XL-fragments
Reporter ions
Ambigous
Unannotated
Noise
rel. intensity

Figure S5. Exemplary fragment ion spectra of three different DSSO-carbamate BSA N-terminal containing cross-links. MS-cleavable fragments are displayed in yellow, b/y ion fragments of the α and β peptides are displayed in blue and red respectively. Highlighted fragments in the three spectra support the N-terminal aspartate as XL site.

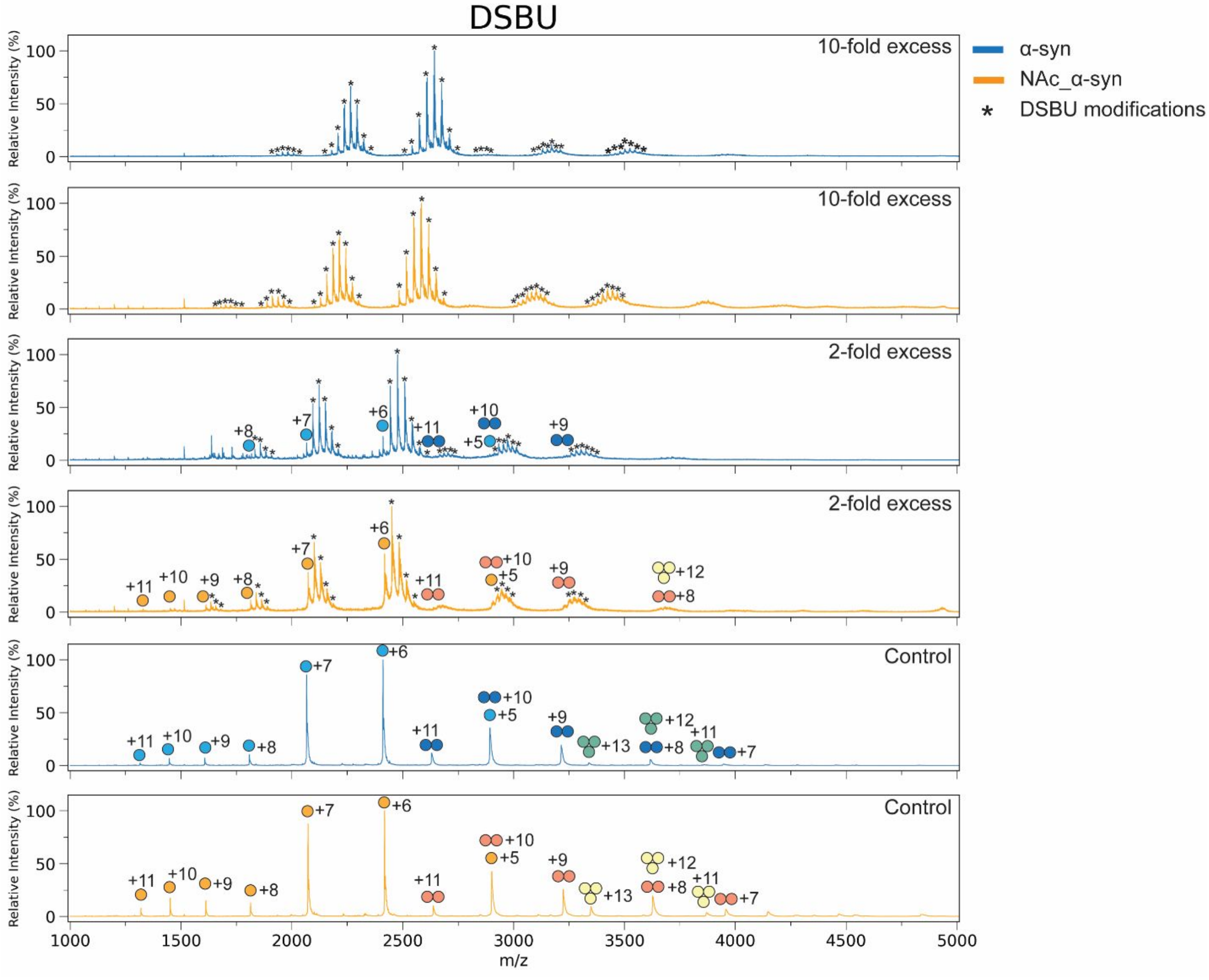

Figure S6. Full native mass spectra of α-syn and NAc_α-syn cross-linked with 2- and 10-fold DSBU molar excess.

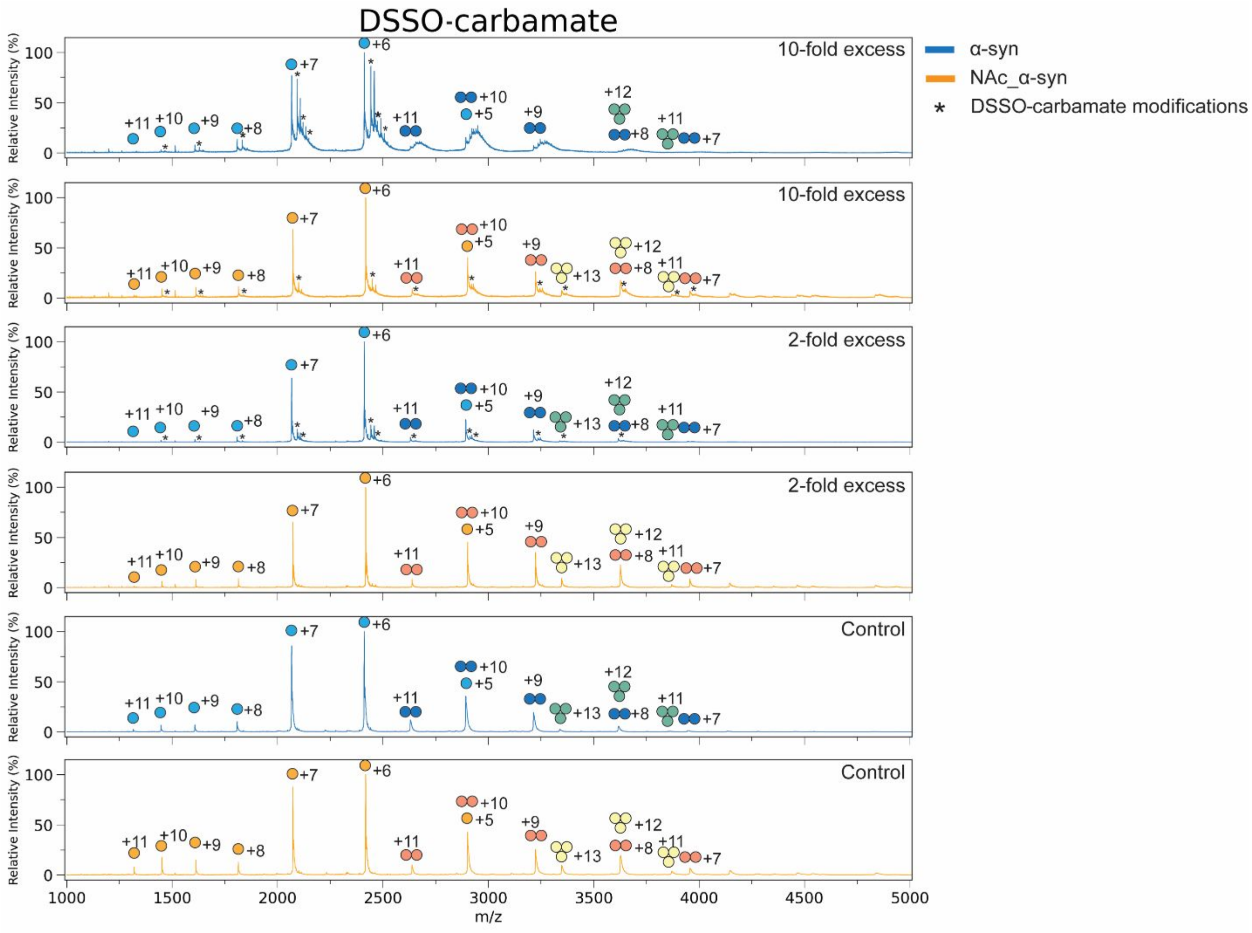

Figure S7. Full native mass spectra of α-syn and NAc_α-syn cross-linked with 2- and 10-fold DSSO-carbamate molar excess.

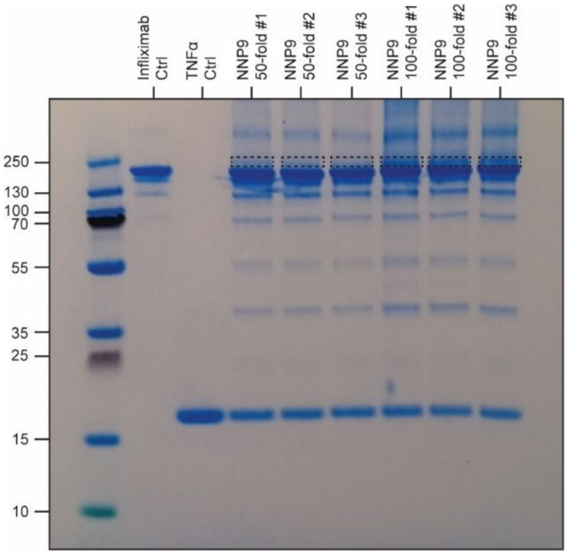

Figure S8. SDS-PAGE for Infliximab and TNFα cross-linked with NNP9 at a concentration of 0.5 mM (50-fold excess over antibody concentration), 1 mM (100-fold excess over antibody concentration). Cross-linked bands of antibody-antigen complexes used for in-gel digestion protocol are highlighted with dashed rectangles.

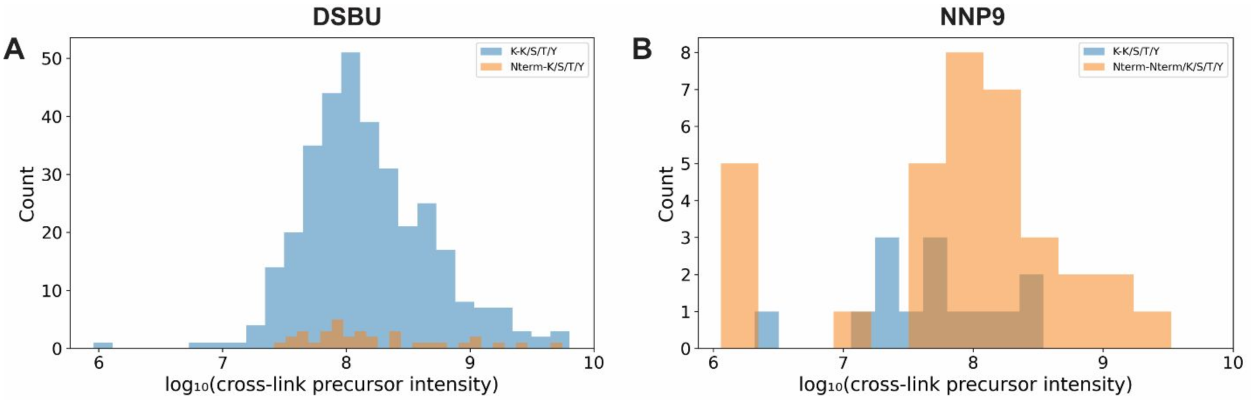

Figure S9. (A) Intensity distribution for DSBU N-terminal containing-links (N term-K/S/T/Y) and lysine containing-links (K-K/S/T/Y) identified in the Infliximab-TNFα dataset. (B) Intensity distribution for NNP9 N-terminal containing-links (Nterm-Nterm/K/S/T/Y) and lysine containing-links (K-K/S/T/Y) identified in the Infliximab-TNFα dataset.

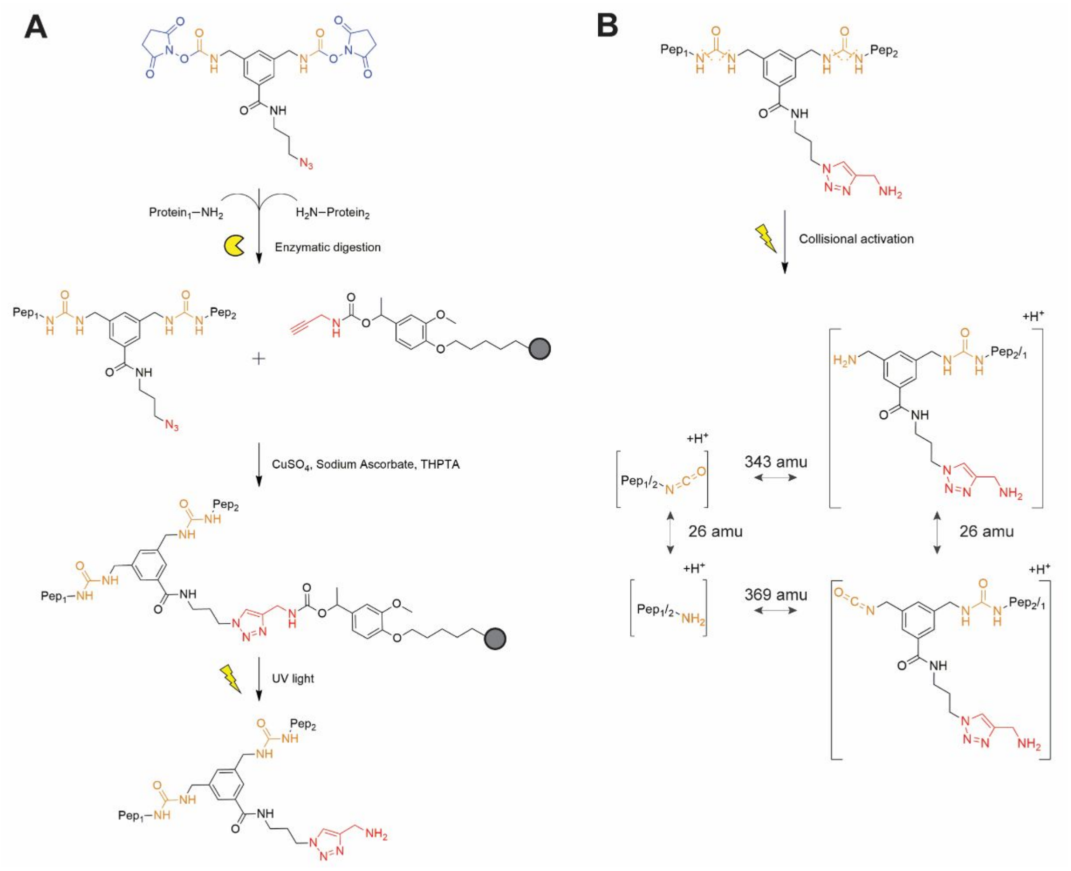

Figure S10. (A) Click-chemistry reaction between the photo-cleavable alkyne beads and the azide tag of NNP9 in the presence of CuSO4, Tris (3-hydroxypropyltriazolylmethyl)amine (THPTA) and sodium ascorbate. After cycloaddition, the clicked NNP9 cross-links are released from the beads under UV light which promotes the cleavage of the carbamate group present on the support. After release, enriched NNP9 cross-links are left with additional atoms compared to NNP9 cross-links before the reaction. (B) NNP9 cross-links after click chemistry enrichment generate as well urea-derived fragments upon higher-energy collisional activation. These diagnostic fragments exhibit defined mass shifts and enable the unambiguous identification of cross-linked peptides.

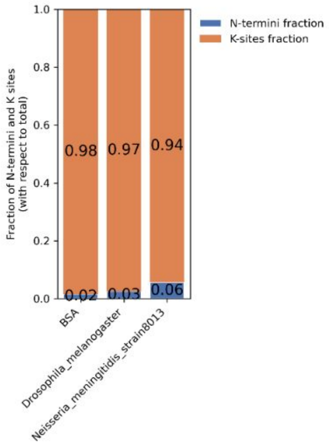

Figure S11. Fraction of N-termini and K-sites for the model protein BSA, Drosophila Melanogaster and Neisseria Meningitidis proteomes. The fraction of N-termini sites is much lower than the one for K-sites (even lower if hydroxyl-containing amino acids would be taken into account).

References

[1] Gotze M., Pettelkau J., Fritzsche R., Ihling C. H., Schafer M., Sinz A., *J Am Soc Mass Spectrom* **2015**, *26*, 83-97.

[2] Gotze M., Iacobucci C., Ihling C. H., Sinz A., *Anal Chem* **2019**, *91*, 10236-10244.

[3] Di Ianni A., Di Ianni A., Cowan K., Barbero L. M., Sirtori F. R., *J Proteome Res* **2024**, *23*, 1049-1061.
